# Personals

**Published:** 1893-11

**Authors:** 


### PERSONALS.

WE are pleased to note that Dr. Ralph E. Smith, after several months stay in New York, has returned to this city and opened an office.

---

DR. Thos. H. Hancock, of Virginia, has located in the city and is associated with Dr. W. C. Jarnagan. We extend to him a cordial welcome.

---

DR. James H. Shorter, of Macon, Ga., was married to Miss Elizabeth McNeil, of Columbus, Ga., on Oct. 11th.

---

DR. E. C. Ripley, recently of this city has removed to Jug Tavern, Ga.

